# The relationship of 3′UTR *HLA‐G14‐bp insertion/deletion* and *+3142 C/G* polymorphisms and soluble HLA‐G expression with gynecological cancers: An updated meta‐analysis

**DOI:** 10.1002/iid3.645

**Published:** 2022-06-06

**Authors:** Kalthoum Tizaoui, Maroua Jalouli, Nadia Boujelbene, Abdel Halim Harrath, Hadda‐Imene Ouzari, Roberta Rizzo, Inès Zidi

**Affiliations:** ^1^ Laboratory Microorganismes and Active Biomolecules, Sciences Faculty of Tunis University of Tunis El Manar Tunis Tunisia; ^2^ Department of Zoology, College of Science King Saud University Riyadh Saudi Arabia; ^3^ Department of Pathology Salah Azaiz Institute Tunis Tunisia; ^4^ Department of Experimental and Diagnostic Medicine, Section Microbiology University of Ferrara Ferrara Italy

**Keywords:** cervical cancer, gynecological cancer, HLA‐G, human papillomavirus infection, meta‐analysis, polymorphism, sHLA‐G

## Abstract

**Objectives:**

Human leukocyte antigen‐G (HLA‐G) is implicated in several cancers and is considered to be an immune checkpoint regulator. We determined the association between polymorphisms in the 3′ untranslated region of *HLA‐G* and soluble HLA‐G (sHLA‐G) expression with gynecological cancers (GCs).

**Methods:**

A meta‐analysis was conducted to examine the association between *HLA‐G14‐bp insertion* (*I*)/*deletion* (*D*) and *+3142C/G* polymorphism in GC and to evaluate sHLA‐G expression

**Results:**

We revealed a significant association between the *+3142C/G* polymorphism and invasive cervical cancer (ICC) based on the allelic model *G* versus *C* (odds ratio [OR] = 0.738, 95% confidence interval [CI] = 0.563–0.966, *p* = 0.027), dominant *GG+GC* versus *CC* (OR = 0.584, 95% CI = 0.395–0.862, *p* = 0.007), and codominant *GG* versus *CC* (OR = 0.527, 95% CI = 0.312–0.891, *p* = 0.017) models, suggesting that the *G* allele and *GG* genotype are protective against ICC. In gynecological precancerous patients with human papillomavirus (HPV) infection, we found that the *14‐bp I/D* under the codominant *DD* versus *DI* model (OR = 0.492, 95% CI = 0.241–1.004, *p* = 0.051) was of borderline significance. Soluble HLA‐G levels were significantly higher in patients compared with healthy controls (standardized mean differences [SMD] = 1.434, 95% CI = 0.442–2.526, *p* = 0.005). Stratification by cancer type revealed that the sHLA‐G levels were significantly increased in cervical cancer (SMD = 4.889, 95% CI = 0.468–9.310, *p* = 0.030) and in subjects of Asian ethnicity (SMD = 4.889, 95% CI = 0.467–9.309, *p* = 0.030).

**Conclusions:**

*HLA‐G14‐bp I/D* and *+3142 C/G* polymorphisms are associated with GC and HPV‐associated cervical cancer. In addition, we found significantly increased sHLA‐G levels in cancer patients. These results provide a basis for further studies in diagnostics and immunotherapy of GC.

## Graphical abstract

*Human leukocyte antigen‐G* (*HLA‐G*)*14‐bp insertion* (*I*)/*deletion* (*D*) and*+3142 C/G* polymorphisms are associated with gynecological cancer (GC) and human papillomavirus‐associated cervical cancer. Soluble HLA‐G is significantly increased in GC.

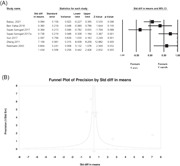

## 1 INTRODUCTION

Human leukocyte antigen‐G (HLA‐G) is a nonclassical major histocompatibility complex class I antigen^1^ encoded by a gene on chromosome 6 at region 6p21.3.^2^ Various physiological factors modulates HLA‐G secretion in fetal tissues,^3^ adult immune‐privileged organs, and cells of hematopoietic lineage.^1^ Furthermore, it is expressed under pathological conditions, including cancer, viral infection, inflammatory diseases, autoimmune diseases, and transplantation.^4^, ^5^, ^6^ In cancer, the expression of HLA‐G is heterogeneous and shows high association with an immunosuppressive microenvironment, advanced tumor stage, poor response to treatment, and prognosis.^7^, ^8^ The expression levels of HLA‐G and its isoforms profiles vary among tumor types, metastasis status, and disease outcome.^9^, ^10^, ^11^ HLA‐G is significantly expressed in ovarian cancer and may directly inhibit the lysis of NK‐92 cells in in vitro experiments.^12^ It helps cancerous ovarian cells in their evasion of host immune‐surveillance.^12^ Thus, it is considered as a potential candidate marker for disease progression.^13^ Cervical carcinoma is a common gynecological malignancy and is the fourth most common cause of mortality from cancer among women worldwide.^14^ Several studies indicate that the infection by the human papillomavirus (HPV) is a risk factor for the development of cervical cancer. A progressive increase in HLA‐G protein expression in the HPV‐infected cervix and cervical carcinoma has been reported.^15^ The progressive upregulation of HLA‐G may be an important factor in the maintenance of HPV conducive to cervical cancer.^15^ Both tumor cells and viruses use the similar strategy to evade from the immune response. Increased HLA‐G expression was revealed in the immune cells of patients infected with cytomegalovirus^16^ and human immunodeficiency virus.^17^ HIV infection appears to increase the HLA‐G production by naïve T CD8+ cells and also the levels of effector and memory cells.^17^ Because of the induction of sHLA‐G by the virus, it is intuitive that antiretroviral therapy would downregulate the HLA‐G production.^18^

Genetic variations in the *HLA‐G* gene, including *14‐bp insertion/deletion* (*I/D*; rs371194629) and *+3142 C/G* (rs1063320), showed significant association with cervical cancer risk^19^ and have been linked to the soluble HLA‐G (sHLA‐G) levels^20^, ^21^, ^22^, ^23^, ^24^ and HLA‐G messenger RNA (mRNA) regulation.^22^, ^23^, ^25^ Interestingly, *HLA‐G* 3′ untranslated region (UTR) alleles were linked to HIV infection in adults^21^, ^26^ and in perinatal HIV transmission.^27^ HIV‐positive women have a higher risk for HPV coinfection, which increases the risk of human cervical cancer.^28^ Both HPV infection^29^, ^30^ and HPV‐associated cervical cancer^31^ showed association with*HLA‐G* gene polymorphisms. Although aberrant HLA‐G expression has been reported to be associated with advanced tumor stage, metastasis status, and poor disease outcome, some discrepancies remain in various cancer types.^32^

The current evidence supports that HLA‐G is involved in cancer; however, discrepancies related to cancer heterogeneity and differences in methods remain. This study aimed to further investigate the role of HLA‐G in gynecological pathologies by analyzing data from existing published reports.

## 2 METHODS

### 2.1 Inclusion and exclusion criteria and data extraction

We searched for published studies involving an association between *HLA‐G* polymorphisms and gynecological cancers in the MEDLINE, Embase, and Cochrane databases (up to December 2021) using Medical Subject Headings and keyword combinations, including “HLA‐G,” “polymorphism,” “gynecological cancer,” and  “neoplasm”. Similarly, we searched for published studies involving the association between sHLA‐G levels and gynecological cancers by using “sHLA‐G,” “level,” “neoplasm,” and “gynecological cancers” as keyword combinations. Furthermore, we identified by a manual search and reviewed additional studies that were not indexed in the MEDLINE, Embase, and Cochrane databases. We considered that studies are eligible if they respond to the following inclusion criteria^1^: evaluation of *HLA‐G* polymorphisms in patients with gynecological cancers and in healthy controls and^2^ the availability of mean and standard deviation (SD) data for sHLA‐G levels in both patients and controls. When only the median and range were reported, we calculated the mean and SD according to the method of Hozo et al.^33^

We excluded studies that (1) included irrelevant, incomplete, and redundant data or (2) were systematic reviews, meta‐analyses, or case reports. For each included study, we extracted the following information: first author, publication year, country, cohort ethnicity, allele, and genetic frequencies of the studied HLA‐G gene polymorphisms, and mean and SD values of sHLA‐G. Two independent reviewers (K. T. and I. Z.) evaluated original studies and extracted the needed data. The meta‐analysis was conducted according to PRISMA guidelines.^34^

### 2.2 Statistical analyses

We conducted a comprehensive meta‐analysis to test the allelic, recessive, dominant, homozygous, and codominant models for *HLA‐G* gene polymorphisms (*14‐bp I/D* and *+3142 C/G*). In case of dichotomous data, the odds ratios (ORs) and 95% confidence intervals (CIs) were calculated. In case of continuous data, standardized mean differences (SMDs) were presented. The heterogeneity and variance between studies were evaluated using the *I* ^2^ and *Tau* ^2^ (*τ* ^2^). The *I* ^2^ values were interpreted according to the Cochrane guidelines.^35^ Heterogeneity was quantified using *I* ^2^, which varied from 0% to 100%, and reflecting the proportion of variation among studies. *I* ^2^ values of 25%, 50%, and 75% were considered low, moderate, and high, respectively. The random‐effects model, testing both sampling errors within the study and variances among studies, is used when heterogeneity among studies is significant (*I* ^2^ > 50%).^36^ The *τ* ^2^ test measures the variance of the true effect sizes and may be used to test the variance of the effect size parameters across studies.^37^ Egger's test of the intercept was employed to estimate the sample size effect, and the two‐tailed *p*_Egger_ value was reported. For the Begg and Mazumdar rank correlation test, we calculated a two‐tailed *p*‐value without continuity correction. The funnel plot is used to measure the study size.^38^ Meta‐analysis and statistical analysis are conducted using the comprehensive meta‐analysis software program (Biostat). A result is considered statistically significant if *p* < 0.05.

## 3 RESULTS

### 3.1 Studies included in the meta‐analysis

**Figure 1:**
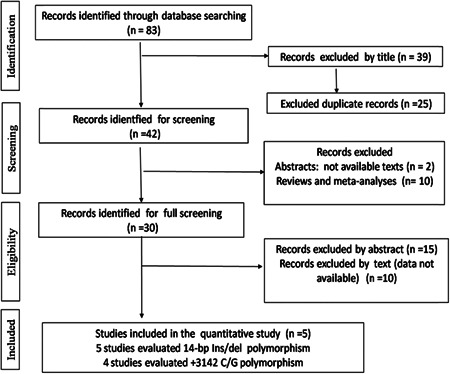
Flow diagram of the systematic review and meta‐analysis literature search results concerning *HLA‐G* polymorphisms in gynecological cancers. HLA‐G, human leukocyte antigen‐G.

We identified 83 eligible studies concerning *HLA‐G* polymorphisms in gynecological cancers. Based on the title and abstract, we selected 42 studies for full‐text analysis. Some studies were excluded because they were reviews or meta‐analyses^10^ and because of unavailable texts^2^ or a lack of relevant data.^25^ Therefore, a total of five articles met our inclusion criteria.^29^, ^31^, ^39^, ^40^, ^41^ The results are summarized in Table 1 and Figure 1.

**Table 1:** Characteristics of studies included in the meta‐analysis: *HLA‐G 14‐bp I/D* and *+3142 C/G* polymorphisms and cervical pathologies.

**Study**	Cancer type	Cancer type	Genotyping method	Country	Ethnicity	Sample size of cases	Sample size of controls	Controls HWE*p*‐value^a^	Controls HWE*p*‐value^b^
Xu et al.^40^	Low‐grade SIL (LSIL)	CIN1 HPV18	PCR	China	Asian	11	185	0.147	0.326
Xu et al.^40^	High‐grade SIL (HSIL)	CIN2 HPV18	PCR	China	Asian	6	185	0.147	0.326
Bortolotti et al.^39^	Invasive cervical cancer (ICC)	ICC	Real‐time PCR	Italy	Caucasian	100	100	0.120	0.073
Bortolotti et al.^39^	Low‐grade SIL (LSIL)	CIN1 HPV high risk	Real‐time PCR	Italy	Caucasian	11	100	0.120	0.073
Bortolotti et al.^39^	Low‐grade SIL (LSIL)	Condyloma	Real‐time PCR	Italy	Caucasian	33	100	0.120	0.073
Yang et al.^41^	Invasive cervical cancer (ICC)	CSCC	PCR‐TaqMan	Taiwan	Asian	317^a^ (315^b^)	400	0.442	0.568
Yang et al.^41^	Invasive cervical cancer (ICC)	CSCC HPV16	PCR‐TaqMan	Taiwan	Asian	171^a^ (170)^b^	400	0.442	0.568
Silva et al.^31^	Invasive cervical cancer (ICC)	ICC	SSP‐PCR	Brazil	Mixed	33	50	0.663	0.112
Silva et al.^31^	High‐grade SIL (HSIL)	HSIL	SSP‐PCR	Brazil	Mixed	22	50	0.663	0.112
Ferguson et al.^29^	Invasive cervical cancer (ICC)	ICC	PCR	Canada	Caucasian	144	833	‐	0.470
Ferguson et al.^29^	High‐grade SIL (HSIL)	CIN2	PCR	Canada	Caucasian	159	833	‐	0.470
Ferguson et al.^29b^	High‐grade SIL (HSIL)	CIN3	PCR	Canada	Caucasian	236	833	‐	0.470

*Note*: HPV high risk: HPV‐16, ‐18, ‐31, ‐33, ‐35, ‐39, ‐45, ‐52, ‐53, ‐56, ‐58, ‐59, ‐66 and ‐70.

Abbreviations: CIN, cervical intraepithelial neoplasia; CSCC, cervical squamous cell carcinoma; HPV, human papillomavirus; HSIL, high‐grade squamous intraepithelial lesions; HWE, Hardy Weinberg equilibrium; ICC, invasive cervical cancer; *I/D, insertion/deletion*; ND, not determined; PCR, polymerase chain reaction; SIL, squamous intraepithelial lesion of the uterine cervix; SSP‐PCR, single specific primer‐PCR.

^a^ Related to the *+3142 C/G* polymorphism.

^b^ Related to the *14‐bp I/D* polymorphism.

**Figure 2:**
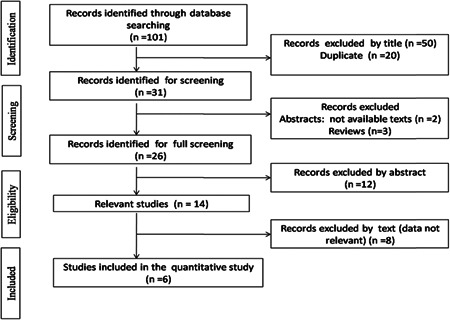
Flow diagram of the systematic review and meta‐analysis literature search results concerning sHLA‐G dosage in gynecological cancers. sHLA‐G, soluble human leukocyte antigen‐G.

For sHLA‐G and gynecological cancers, 101 studies were identified. Of these, 31 were selected based on the title and abstract for full‐text analysis. Studies were excluded as they were reviews^3^ and because of unavailable text,^2^ irrelevant data,^12^ and lack of data.^9^ This resulted in a total of six articles that met our inclusion criteria.^42^, ^43^, ^44^, ^45^, ^46^, ^47^ Selected study details are summarized in Table 2 and Figure 2.

**Table 2:** Characteristics of studies included in the meta‐analysis: sHLA‐G and gynecological cancers.

Study	Disease	Quantification method	Cases age (years)	Country	Ethnicity	Biologic liquid	Cases		Controls		Units
			Mean ± SD or median (range)				Sample size of cases	Mean ± SD	Sample size of controls	Mean ± SD	
Babay et al.^42^	Ovarian cancer	ELISA/mAb: MEM‐G9*(Exbio)*	54.77 ± 13.69	Tunisia	Caucasian	Plasma	79	2.13 ± 9.94	80	1.4 ± 7.24	ng/ml
Ben Yahia et al.^43^	Endometrial cancer	ELISA/mAb: MEM‐G9*(Exbio)*	62.25 ± 9.55	Tunisia	Caucasian	Plasma	40	2.352 ± 8.911	45	0.145 ± 0.684	ng/ml
Sipak‐Szmigiel et al.^45^	Ovarian cancer	ELISA kit*(Bio Vendor Laboratory Medicine)*	73	Poland	Caucasian	Serum	38	9.967 ± 2.233	54	11.225 ± 4.100	U/ml
Sipak‐Szmigiel^45^	Ovarian cancer	ELISA kit *(Bio Vendor Laboratory Medicine)*	73	Poland	Caucasian	Ascite	38	38.765 ± 10.350	54	31.708 ± 9.018	U/ml
Sun et al.^46^	Cervical cancer	ELISA kit*(AMEKO)*	56.04 ± 14.53	China	Asian	Ascite	2	21.955 ± 2.864	30	12.467 ± 3.678	μg/L
Zheng et al.^47^	Cervical cancer	*ELISA kit (Exbio)*	*45 (20–73)*	China	Asian	Plasma	80	206.950 ± 25.133	20	45.963 ± 4.353	U/ml
Rebmann et al.^44^	Ovarian cancer	ELISA/mAb (NS)	NS	Germany	Caucasian	Plasma	22	33.200 ± 18.762	92	22.600 ± 17.265	ng/ml

*Note*: Italics in column 3 correspond to the manufacturer.

Abbreviations: mAb, monoclonal antibody of detection; NS, not specified.

### 3.2 Meta‐analysis of the association between *HLA‐G +3142 C/G* and *14‐bp I/D* polymorphisms and their haplotypes with gynecological cancers

**Figure 3:**
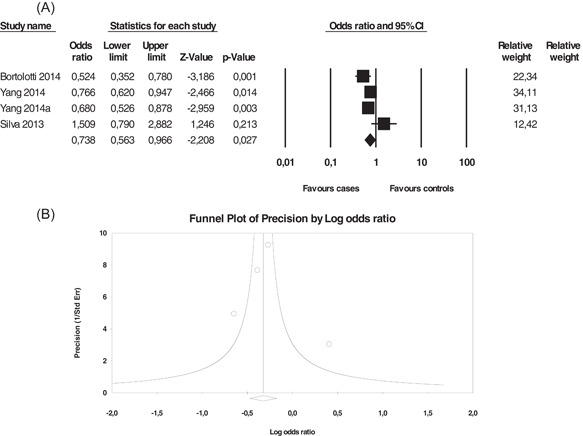
Association between *HLA‐G +3142 C/G* polymorphism and invasive cervical cancer. (A) Forest plot under allelic model (*G* vs. *C*), (B) funnel plot under allelic model. Forest plot shows the odds ratio and respective 95% confidence intervals for the different studies included in the meta‐analysis. For each study in the forest plot, the area of the black square is proportional to study weight and the horizontal bar represents the 95% confidence interval. *Z*‐score: the standardized expression of a value in terms of its relative position in the full distribution of values. CI, confidence interval. Funnel plot assesses the presence or absence of publication bias. HLA‐G, human leukocyte antigen‐G.

Meta‐analysis demonstrated a significant association between the *+3142 C/G* polymorphism and invasive cervical cancer (ICC) under the allelic model, *G* versus *C* (OR = 0.738, 95% CI = 0.563–0.966, *p* = 0.027) (Figure 3A), dominant *GG* + *GC* versus *CC* (OR = 0.584, 95% CI = 0.395–0.862, *p* = 0.007), and codominant *GG* versus *CC* (OR = 0.527, 95% CI = 0.312–0.891, *p* = 0.017) models, indicating that the*G* allele and*GG* genotype may represent a protective allele and genotype against ICC. The *+3142 C/G* polymorphism did not exhibit an association with low‐grade squamous intraepithelial lesions (LSIL) or high‐grade squamous intraepithelial lesions (HSIL). Both conditions were classified as squamous intraepithelial lesions of the uterine cervix (Table 3). Heterogeneity was moderate to high in the ICC and LSIL groups (*I* ^*2*^ > 50%). In the HSIL group, only two studies were included, and therefore, heterogeneity was not detected (*I* ^2^ = 0%). The variance observed in the forest plot reflects a difference in the true effect sizes rather than sampling errors, suggesting calculations in the random‐effects model. Egger and Begg's*p*‐values were not significant (*p*‐value two‐tailed > 0.05), suggesting the absence of the sample size effect for all genetic models of ICC. A funnel plot was symmetric, revealing the absence of publication bias (Figure 3B). These results confirm the robustness of the results revealed by this meta‐analysis.

**Table 3:** Meta‐analysis of the association between *HLA‐G +3142 C/G* polymorphism and cervical pathologies under random effects model.

Genetic model		Gynecologic pathologies	*N*	Odds ratio			Heterogeneity			*p*_Egger_	*p*_Begg_
				OR	95% CI	*p*_OR_	*I* ^2^ (%)	*τ* ^2^	*p*_H_		
*HLA‐G *+*3142 C/G* (*N* = 9)											
Allelic											
*G vs. C*		LSIL	3	1.584	0.234–10.720	0.637	91.5	2.590	**0.000**	0.056	0.117
		HSIL	2	1.367	0.731–2.559	0.328	0	0	0.855	‐	‐
		ICC	4	0.738	0.563–0.966	**0.027**	62.4	0.044	**0.046**	0.670	1.000
Dominant											
*GG* + *GC vs. CC*		LSIL	3	1.571	0.124–19.970	0.728	78.8	3.750	**0.016**	**0.003**	0.602
		HSIL	2	2.003	0.549–7.307	0.293	0	0	0.650	‐	‐
		ICC	4	0.584	0.395–0.862	**0.007**	47.1	0.070	0.128	0.104	0.174
Recessive											
*GG vs. GC* + *CC*		LSIL	3	1.425	0.207–9.828	0.719	85.9	2.500	**0.001**	0.229	0.117
		HSIL	2	1.288	0.534–3.107	0.574	0	0	0.606	‐	‐
		ICC	4	0.723	0.454–1.152	0.172	71.3	0.148	**0.015**	0.725	1.000
Homozygous											
*GG* + *CC vs. GC*		LSIL	3	1.357	0.735–2.505	0.330	0	0	0.876	0.328	0.117
		HSIL	2	0.867	0.364–2.068	0.748	0	0	0.440	‐	‐
		ICC	4	1.052	0.719–1.540	0.795	62.4	0.088	**0.046**	0.320	0.497
Codominant OR1											
*GG* vs. *CC*		LSIL	3	1.806	0.067–48.958	0.726	83.8	7.012	**0.002**	**0.001**	0.117
		HSIL	2	2.135	0.535–8.526	0.283	0	0	0.855	‐	‐
		ICC	4	0.527	0.312–0.891	**0.017**	60.5	0.158	0.055	0.620	1.000
Codominant OR2											
*GC* vs. *CC*		LSIL	3	1.116	0.177–7.033	0.907	52.6	1.450	0.121	**0.037**	0.602
		HSIL	2	1.895	0.478–7.508	0.363	0	0	0.508	‐	‐
		ICC	4	0.648	0.404–1.040	0.073	57	0.122	0.073	0.091	0.174
Codominant OR3											
*GG* vs. *GC*		LSIL	3	1.274	0.345–4.707	0.716	67.4	0.898	**0.047**	0.395	0.117
		HSIL	2	1.094	0.429–2.791	0.851	0	0	0.501	‐	‐
		ICC	4	0.817	0.488–1.368	0.442	72.7	0.186	**0.012**	0.490	0.174

*Note*: Bold significant *p*‐value (≤0.05).

Abbreviations: CI, confidence interval; HLA‐G, human leukocyte antigen‐G; HSIL, high‐grade squamous intraepithelial lesions; ICC, invasive cervical cancer; LSIL, low‐grade squamous intraepithelial lesions; N, number of studies; OR, odds ratio; *p*_Begg_, *p*‐value associated to Begg and Mazumdar rank correlation test (two‐tailed) without continuity correction; *p*_Egger_, *p*‐value associated to Egger's test (two‐tailed); *p*_H_, *p*‐value associated to heterogeneity.

**Figure 4:**
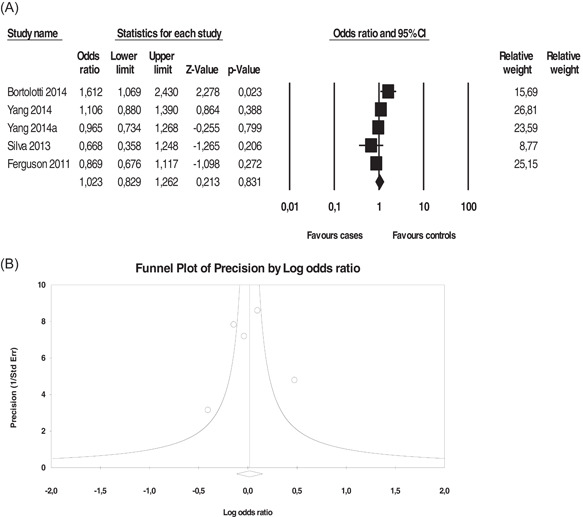
Association between *HLA‐G 14‐bp I/D* polymorphism and invasive cervical cancer. (A) Forest plot under allelic model (*D* vs. *I*), (B) funnel plot under allelic model. Forest plot shows the odds ratio and respective 95% confidence intervals for the different studies included in the meta‐analysis. For each study in the forest plot, the area of the black square is proportional to study weight and the horizontal bar represents the 95% confidence interval. *Z*‐score, the standardized expression of a value in terms of its relative position in the full distribution of values. CI, confidence interval. Funnel plot assesses the presence or absence of publication bias. HLA‐G, human leukocyte antigen‐G.

The *14‐bp I/D* did not show a significant association in the study models (Figure 4A, Table 4). We detected substantial heterogeneity, particularly for ICC; however, LSIL and HSIL showed low to moderate heterogeneity. We did not detect a publication bias using Egger and Begg's tests as well as a funnel plot (Figure 4B).

**Table 4:** Meta‐analysis of the association between *HLA‐G 14‐bp I/D* polymorphism and cervical pathologies under random effects model.

Genetic model		Gynecologic pathologies	*N*	Odds ratio			Heterogeneity			*p*_Egger_	*p*_Begg_
				OR	95% CI	*p*_OR_	*I* ^2^ (%)	*τ* ^2^	*p*_H_		
*HLA‐G 14‐bp I/D* (*N* = 12)											
Allelic											
*D vs. I*		LSIL	3	1.039	0.680–1.589	0.859	0	0	0.646	0.767	0.602
		HSIL	4	0.910	0.658–1.259	0.571	62.7	0.056	**0.045**	0.186	0.174
		ICC	5	1.023	0.829–1.262	0.831	54.2	0.029	0.068	0.976	1.000
Dominant											
*DD* + *DI vs. II*		LSIL	3	1.997	0.395–10.100	0.403	42.841	0.886	0.174	0.471	0.602
		HSIL	4	0.962	0.586–1.580	0.879	52	0.117	0.100	0.143	0.174
		ICC	5	0.802	0.505–1.271	0.347	67.2	0.175	**0.016**	0.154	0.142
Recessive											
*DD vs. DI* + *II*		LSIL	3	0.642	0.336–1.227	0.180	1.4	0.005	0.363	0.444	0.602
		HSIL	4	0.906	0.616–1.334	0.617	44.3	0.060	0.145	0.247	0.174
		ICC	5	1.222	0.962–1.550	0.100	34.5	0.025	0.191	0.215	0.624
Homozygous											
*DD* + *II vs. DI*		LSIL	3	0.482	0.152–1.533	0.217	67.6	0.703	0.046	0.064	0.117
		HSIL	4	0.898	0.725–1.111	0.322	0	0	0.860	0.670	0.497
		ICC	5	0.856	0.350–2.092	0.733	95.1	0.964	**0.000**	0.859	1.000
Codominant OR1											
*DD vs. II*		LSIL	3	1.536	0.428–5.510	0.511	4.6	0.063	0.351	0.643	0.602
		HSIL	4	0.858	0.447–1.644	0.643	61.3	0.224	0.051	0.170	0.174
		ICC	5	0.452	0.144–1.422	0.175	94.5	1.564	**0.000**	0.567	0.624
Codominant OR2											
*DI vs. II*		LSIL	3	2.450	0.370–16.229	0.353	53.6	1.491	0.116	0.337	0.117
		HSIL	4	1.139	0.845–1.537	0.393	1.442	0.002	0.385	0.148	0.174
		ICC	5	0.719	0.411–1.257	0.247	73.3	0.282	**0.005**	0.054	0.142
Codominant OR3											
*DD vs. DI*		LSIL	3	0.542	0.223–1.321	0.178	40.1	0.251	0.188	0.189	0.117
		HSIL	4	0.916	0.692–1.211	0.537	11.9	0.011	0.333	0.306	0.497
		ICC	5	0.467	0.134–1.626	0.231	96.8	1.923	**0.000**	0.657	0.327

*Note*: Bold significant *p*‐value (≤0.05).

Abbreviations: CI, confidence interval; HLA‐G, human leukocyte antigen‐G; HSIL, high‐grade squamous intraepithelial lesions; ICC, invasive cervical cancer; LSIL, low‐grade squamous intraepithelial lesions; N, number of studies; OR, odds ratio; *p*_Begg_, *p*‐value associated to Begg and Mazumdar rank correlation test (two‐tailed) without continuity correction; *p*_Egger_, *p*‐value associated to Egger's test (two‐tailed); *p*_H_, *p*‐value associated to heterogeneity.

**Figure 5:**
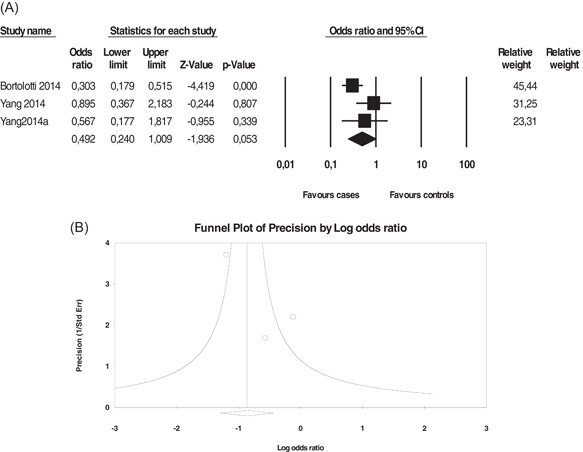
Association between *HLA‐G Del/G* haplotype and invasive cervical cancer (A) forest plot, (B) funnel plot. Forest plot shows the odds ratio and respective 95% confidence intervals for the different studies included in the meta‐analysis. For each study in the forest plot, the area of the black square is proportional to study weight and the horizontal bar represents the 95% confidence interval. *Z*‐score, the standardized expression of a value in terms of its relative position in the full distribution of values. CI, confidence interval. Funnel plot assesses the presence or absence of publication bias. HLA‐G, human leukocyte antigen‐G.

Haplotype analysis, including *14‐bp I/D* and *+3142 C/G* polymorphisms, revealed borderline associations for the *Del/G* haplotypes in ICC (OR = 0.492, 95% CI = 0.240–1.009, *p* = 0.053) (Figure 5A). However, only three studies were included, and more studies and stratification analyses should reveal new and strong associations (Table 5). Heterogeneity was absent for the *I/G* haplotype but moderate to high for the *D/G*, *D/C*, and *I/C* haplotypes. We did not detect any publication bias using a funnel plot (Figure 5B).

**Table 5:** Meta‐analysis of the Association between *HLA‐G* haplotypes and cervical pathologies under random effects models.

Genetic model	Gynecologic pathologies	*N*	Odds ratio			Heterogeneity			*p*_Egger_	*p*_Begg_
			OR	95% CI	*p*_OR_	*I* ^2^ (%)	*τ* ^2^	*P*_H_		
*HLA‐G 14‐bp I/D/HLA‐G *+*3142 C/G haplotypes* (*N* = 7)										
*I/G*	LSIL	3	1.006	0.637–1.590	0.980	0	0	0.369	0.035	0.602
	ICC	3	1.303	0.949–1.788	0.102	0	0	0.490	0.009	0.117
*D/G*	LSIL	3	0.999	0.210–4.753	0.999	88.8	1.686	0.000	0.155	0.602
	ICC	3	0.492	0.240–1.009	0.053	55.3	0.223	0.107	0.407	0.602
*D/C*	LSIL	3	0.592	0.135–2.603	0.488	86.1	1.455	0.001	0.083	0.602
	ICC	3	1.097	0.438–2.746	0.843	86.3	0.562	0.001	0.141	0.602
*I/C*	LSIL	2	1.903	0.250–14.496	0.534	53.1	1.337	0.144	‐	‐

*Note*: Bold significant *p*‐value (≤0.05).

Abbreviations: CI, confidence interval; HLA‐G, human leukocyte antigen‐G; HSIL, high‐grade squamous intraepithelial lesions; ICC, invasive cervical cancer; LSIL, low‐grade squamous intraepithelial lesions; N, number of studies; OR, odds ratio; *p*_Begg_, *p*‐value associated to Begg and Mazumdar rank correlation test (two‐tailed) without continuity correction; *p*_Egger_, *p*‐value associated to Egger's test (two‐tailed); *p*_H_, *p*‐value associated to heterogeneity.

### 3.3 Association of sHLA‐G with gynecological cancers

**Figure 6:**
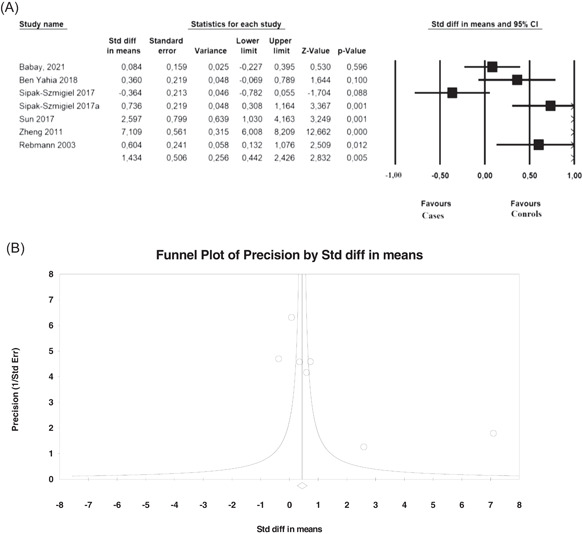
Association of sHLA‐G and gynecological cancers. (A) Forest plot and (B) funnel plot of overall population. sHLA‐G, soluble human leukocyte antigen‐G.

Soluble HLA‐G levels were significantly higher in gynecological cancers compared with the controls (SMD = 1.434, 95% CI = 0.442–2.426, *p* = 0.005) (Figure 6A). Stratification by cancer type revealed that the sHLA‐G levels were significantly increased in cervical cancer (SMD = 4.889, 95% CI = 0.468–9.310, *p* = 0.030; Table 6); however, ovarian cancer did not exhibit significantly increased levels. Stratification by ethnicity revealed that Asian patients have significantly increased sHLA‐G levels (SMD = 4.889, 95% CI = 0.467–9.309, *p* = 0.030; Table 6); however, Caucasians did not exhibit increased sHLA. The plasma/serum samples showed significantly higher sHLA‐G levels (SMD = 1.428, 95% CI = 0.142–2.715, *p* = 0.030; Table 6), whereas peritoneal fluids (ascites) did not show an increase. Because the number of included studies was small (*N* = 2), more larger cohorts are required to confirm the conclusion of the present study. All calculations were performed with the random‐effects model, because heterogeneity was high (*I* ^2^ > 75%). The high heterogeneity detected among the studies in the overall analysis (*I* ^2^ = 96.5%) could be explained by the heterogeneity of cancers in addition to differences in the sHLA‐G detection methods (Table 6). No bias of publication was detected (Figure 6B).

**Table 6:** Meta‐analysis of the association of sHLA‐G with gynecological cancers under random effects models.

	*N*	Standardized mean differences				Heterogeneity			*p*_Egger_	*p*_Begg_
		SMD	SEM	95% CI	*p*_SMD_	*I* ^2^ (%)	*τ* ^2^	*P*_H_		
Overall population	7	1.434	0.506	0.442–2.426	**0.005**	96.5	1.647	**0.000**	**0.046**	0.051
Cervical cancer	2	4.889	2.256	0.468–9.310	**0.030**	95.3	9.702	**0.000**	‐	‐
Ovarian cancer	4	0.255	0.239	−0.214–0.724	0.286	81.6	0.185	**0.000**	0.607	0.497
Caucasian	5	0.275	0.189	−0.098–0.645	0.149	76.1	0.135	**0.002**	0.488	0.327
Asian	2	4.889	2.256	0.467–9.309	**0.030**	95.3	9.702	**0.000**	‐	‐
Ascite	2	1.508	0.917	−0.289–3.304	0.100	80.2	1.388	**0.025**	‐	‐
Plasma/serum	5	1.428	0.656	0.142–2.715	**0.030**	97.5	2.061	**0.0002**	**0.038**	**0.050**

*Note*: Bold significant *p*‐value (≤0.05).

Abbreviations: CI, confidence interval; HLA‐G, soluble human leukocyte antigen‐G; N, number of studies; NA, not applicable; OR, odds ratio; *p*_Begg_, *p*‐value associated to Begg and Mazumdar rank correlation test (two‐tailed) without continuity correction; *p*_Egger_, *p*‐value associated to Egger's test (two‐tailed); *p*_H_, *p*‐value associated to heterogeneity; *p*_SMD_, *p*‐value associated to SMD; R, random effects model; SEM, standard errors of the mean; SMD, standardized mean differences.

## 4 DISCUSSION

Gynecological pathologies, particularly gynecological cancers, are multifactorial disorders in which genetic factors constitute strong determinants. HLA‐G, a potent inhibitory checkpoint of immune cell function, is highly expressed in gynecological cancers. The membranous HLA‐G is significantly expressed in cancers and plays a significant role in their diagnosis and progression.^48^, ^49^, ^50^ Moreover, the vesicular‐bound HLA‐G was associated with a high risk of ovarian cancer progression.^51^ To the best of our knowledge, this is the first meta‐analysis investigating the association of 3′UTR *HLA‐G* gene polymorphisms (*14‐bp I/D* and *+3142 C/G*) and sHLA‐G with gynecological pathologies. By pooling several eligible studies, the meta‐analysis provided accurate data by increasing the statistical power and analysis resolution. In the present study, we evaluated eligible studies that included 1243 patients for *HLA‐G 14‐bp I/D*, 1240 patients and *HLA‐G +3142 C/G*, along with 4069 controls. Our data revealed a link between ICC and the *+3142 C/G* polymorphism. However, squamous intraepithelial lesions classified as precancerous lesions did not exhibit an association. We demonstrated that the *G* allele and *GG* genotype are likely protective against ICC under different contrast models (allele, dominant, and codominant models). The presence of the *G* allele was proposed to enhance the affinity of HLA‐G mRNA for microRNAs, thus increasing the instability of the HLA‐G transcripts,^52^ which may explain this protection. Indeed, ICC patients with one or two exemplary*G* alleles may exhibit low HLA‐G expression and reduced immune tolerance associated with the HLA‐G molecule.

The meta‐analysis conducted for the *HLA‐G 14‐bp I/D* did not reveal an association with precancerous lesions or with ICC (Table 4). However, we reported that the *Del/G* haplotypes exhibited a borderline association with ICC. *Del/G* is proposed as a potential protective haplotype; however, more larger studies are required to corroborate these findings.

The current meta‐analysis conducted on 299 cases and 375 controls revealed increased levels of sHLA‐G in all gynecological cancers (SMD = 1.434, 95% CI = 0.442–2.426, *p* = 0.005) and, particularly, in cervical cancer (SMD = 4.889, 95% CI = 0.468–9.310, *p* = 0.030). After stratification by ethnicity, the association of increased sHLA‐G expression with gynecological cancers was maintained only for the Asian population (*p* = 0.030). Our findings support sHLA‐G as a key biomarker for gynecological cancers. It plays an important role as a powerful immune regulator implicated in immune tolerance and the inhibition of immune cell function.

Although limited publication bias proves the robustness of our analyses, this meta‐analysis is the first to investigate *HLA‐G* polymorphisms and sHLA‐G expression in gynecological cancers. Our results provide two parallel lines of evidence exploring both the sHLA‐G levels and certain *HLA‐G* 3′UTR polymorphisms in patients with gynecological pathologies. HLA‐G, which is highly expressed during gynecological carcinogenesis, might be an excellent molecular target for immunotherapy. More studies are needed to decipher the pathways associated with cervix infections and cervical cancer. A recent meta‐analysis by Moossavi et al.^53^ has investigated *HLA‐G +3142 C/G* genetic variant and the risk of human papillomavirus infection, but they did not found any significant association. However, more studies still needed to confirm this finding since cancer is a multifactorial disease, and persistent viral infections would increase considerably cancer susceptibility and/or progression.

Nevertheless, there are some limitations associated with the present study that require consideration. The first is the limited number of eligible studies available for analysis. Second, there was a poor representation of some ethnicities, such as Africans, Middle Eastern populations, and Americans. Third, we detected significant heterogeneity among the studies and subgroups. This is not surprising since discrepancies among and within different tumor types with respect to sHLA‐G expression profiles have been observed in various cancers.^54^ The discrepancies may reflect differences between assessment methods, which include a wide variation of experimental protocols.

## 5 CONCLUSION

This meta‐analysis of the association between *HLA‐G* 3′UTR polymorphisms and sHLA‐G expression with gynecological cancers revealed (i) a significant association of *HLA‐G +3142 C/G* with reduced susceptibility to cervical pathologies and (ii) high sHLA‐G levels in patients with gynecological cancers, which support an important role for *HLA‐G* polymorphisms and sHLA‐G expression in the pathogenesis of gynecological cancers.

## AUTHOR CONTRIBUTIONS

*Data collection, Data analysis and interpretation, Drafting of manuscript*: Kalthoum Tizaoui. *Medical validation, Critical data interpretation, Critical revision*: Nadia Boujelbene. *Critical data interpretation, Critical revision*: Maroua Jalouli, Abdel Halim Harrath, Hadda‐Imene Ouzari, and Roberta Rizzo.*Study conception and design, supervision, Data analysis, Drafting of manuscript, Critical revision*: Inès Zidi.

## CONFLICT OF INTEREST

The authors declare no conflict of interest.

## Data Availability

The data that support the findings of this study are available from the corresponding author upon reasonable request.
